# *Glomus mosseae* Inoculation Improves the Root System Architecture, Photosynthetic Efficiency and Flavonoids Accumulation of Liquorice under Nutrient Stress

**DOI:** 10.3389/fpls.2017.00931

**Published:** 2017-06-07

**Authors:** Meilan Chen, Guang Yang, Ye Sheng, Pengying Li, Hongyan Qiu, Xiuteng Zhou, Luqi Huang, Zhi Chao

**Affiliations:** ^1^State Key Laboratory of Dao-Di Herbs, National Resource Center for Chinese Materia Medica, China Academy of Chinese Medical SciencesBeijing, China; ^2^School of Public Health, Ningxia Medical UniversityYinchuan, China; ^3^School of Traditional Chinese Medicine, Southern Medical UniversityGuangzhou, China

**Keywords:** *Glycyrrhiza uralensis*, arbuscular mycorrhizal fungus, nutrient, root system architecture, photosynthesis, flavonoids

## Abstract

The poor quality and low productivity of cultivated liquorice (*Glycyrrhiza uralensis*) continues to put pressure on wild plant populations. As arbuscular mycorrhizal fungi are known to support plant growth and in some cases even to enhance the accumulation of valuable molecules in the plant, the effect of *Glomus mosseae* on the growth and active ingredient contents was evaluated in liquorice plants grown under nutrient deficiency. We created a nutrient-deficient environment by mixing paddy soil, washed river sand, and pumice at a ratio of 1:5:1. Our results showed that the inoculation of pot-grown liquorice plants with *G. mosseae* significantly increased the shoot and root biomass (by 25- and 17-folds, respectively) and the contents of glycyrrhizic acid, liquiritin, isoliquiritin, and isoliquiritigenin in the main root (by 1.6-, 4.8-, 6.5-, and 4.4-folds, respectively). Both isoliquiritin and isoliquiritigenin were detectable in the lateral roots of the plants inoculated with *G. mosseae*, but not in plants without *G. mosseae* inoculation. *G. mosseae* inoculation improved the features of the root system and increased photosynthetic efficiency of liquorice. The uptake of P and K by liquorice increased when *G. mosseae* was inoculated, leading to the depletion of these macronutrients in the soil; *G. mosseae* also improved the availability of Mg, Cu, Zn, and Mn. Based on these results, we concluded that the inoculation of liquorice plants with *G. mosseae* is beneficial, particularly for those grown in nutrient-deficient soil, and such positive effect is related to the improvement of the root system and an increased photosynthetic efficiency.

## Introduction

Liquorice (*Glycyrrhiza uralensis* Fisch.) is a leguminous species grown widely in the northern, north-eastern, and north-western regions of China^1^. As a hardy plant well adapted to soils of low fertility, liquorice has been shown to restore degraded soils in arid and semi-arid regions of the country (Pan et al., 2006; Liu et al., 2007). The major value of liquorice, however, lies in the valuable compounds found in its roots, some of which are associated with positive health effects and have substantial economic and pharmacological values (Hayashi and Sudo, 2009). The increasing demand for liquorice has caused much pressure on its wild populations (Wang et al., 2003; Hodge et al., 2009), and over the past 30 years, attempts have been made to cultivate it as a crop out of both economic and ecological considerations, but both the yield and quality of cultivated liquorice has remained insufficient (Wei et al., 2003).

Liquorice cultivation is targeted at nutrient-poor soils. In order to obtain a satisfactory yield and quality, intensive fertilization is indispensable (Fu et al., 2013; Fan et al., 2016). But massive and long-term application of chemical fertilizer will lead to deterioration of soil physical and chemical properties and environmental pollution (Zhang et al., 2010). Arbuscular mycorrhizal fungi (AMF) as a biofertilizer provide us with a substitute solution. Already known advantages of symbiosis with AMF include: the promotion of vegetative growth (Huang et al., 2010; Khabou et al., 2014), secondary metabolite content (Sarkar et al., 2015; Urcoviche et al., 2015), and nutrient acquisition of plant (Weisany et al., 2016); the improvement of soil conditions for the host plants by improving the soil structure and soil aggregate stability (Piotrowski et al., 2004; Van der Heijden et al., 2006); and the contribution to the ecosystem stability (Feddermann et al., 2010).

It was reported that inoculation of *Glomus mosseae* (=*Funneliformis mosseae*) (Schüßler and Walker, 2010) and *G. versiforme*, alone or in combination, improved *G. uralensis* plant growth during early and late growth stages in comparison with the control; In addition, mycorrhiza formation enhanced the glycyrrhizic acid concentration in roots, but resulted in a considerable reduction of the root oxidase activity (Liu et al., 2007). Similarly, *G. mosseae* and *G. intraradices* could stimulate accumulation of glycyrrhizic acid in plantlets of another *Glycyrrhiza* species, *G. glabra*; and *G. mosseae* was more effective (Orujeia et al., 2013).

**FIGURE 1:**
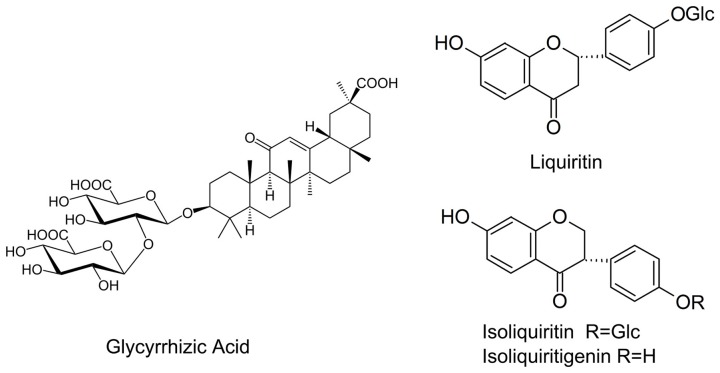
Structure of some bioactive compounds in liquorice.

Flavonoids is another another group of bioactive constituents in *G. uralensis*, of which liquiritin, liquiritgenin, isoliquirtin, isoliquiritigenin, and licochalcone (**Figure 1**) represent the major ones (Asl and Hosseinzadeh, 2008; Fujii et al., 2014). Effects of AMF on the flavonoids in liquorice under nutrient deficiency have not been thoroughly explored. Nor is it clear whether the presence of AMF has any influence on important factors affecting plant growth status such as photosynthetic efficiency and root architecture of the host plant.

In this study, we created a nutrient-deficient environment for liquorice growth by mixing paddy soil, washed river sand, and pumice, and aimed to evaluate the effects of inoculating *G. uralensis* with the AMF species *G. mosseae* on the accumulation of active ingredients, both glycyrrhizic acid and the flavonoids, in root of the plant under such a nutrient stress. We also examined the effect of the treatment on detailed morphological changes of the host plant roots, its utilization of mineral nutrients in soil, and photosynthetic efficiency of the aerial parts.

## Materials and Methods

### Plant and AMF Materials and Cultivation

Liquorice seeds were purchased from a retailer in Anguo Market of Chinese Traditional and Herbal Drugs, which is located at Hebei Province in North China. After germination, the seedlings were identified as *G. uralensis* by the authors. *G. mosseae* was obtained from Professor Honggang Wang (Beijing Chinese Academy of Agricultural Sciences, China). The fungi were propagated using sorghum (*Sorghum bicolor*) as the host, and the infected roots, hyphae, spores, and substrates were collected. The pot-based plant culture involved growing liquorice plant in a 1:5:1 mixture of paddy soil, washed river sand, and pumice. The culture medium contained organic matter (0.49 g/kg), total N (3.85 g/kg), total P (8.43 g/kg), available P (2.27mg/kg), total K (28.43 g/kg), available K (8.71 mg/kg), available Zn (0.07 mg/kg), available Mn (0.74 mg/kg), available Fe (1.6mg/kg), and available Cu (0.13mg/kg), with a pH value of 8.7. The medium was passed through a 2-mm mesh sieve and autoclaved for 2 h. Each pot was filled with 800 g medium plus 40 g (equivalent to ∼500 spores) of either live or heat-killed (autoclaved at 121°C for 30 min) inoculum. Prior to autoclaving, the inoculum was washed and filtered through a 20 μm mesh twice to remove AMF propagules. This filtrate was added to all CK pots to create a uniform microbial community in all treatments (Karasawa et al., 2012). Liquorice seeds were surface-sterilized by immersion in 0.5% (*V/V*) NaClO for 10 min, followed by a wash in tap water for 30 min.

### Experimental Design

The experiment consisted of 6 pots containing live inoculum (GM) and 6 containing heat-killed inoculum (CK) randomly arranged in a greenhouse (Liu et al., 2007; Karasawa et al., 2012; Orujeia et al., 2013). The liquorice seeds were sown directly into the pot and thinned to two seedlings per pot 1 week after seedling emergence. The pots were weighed three times per week and watered to maintain a field capacity of the medium at around 80%. After 6 months, seedlings were fertilized with 20% Hoagland’s solution which do not contain P nutrient (Hoagland and Arnon, 1950). The plants were harvested 1 year after sowing, at which point the fresh weights of the shoots and roots were recorded, and root system measurement was done. Some fresh root samples were used to visualize AMF colonization. Macro- and micronutrients in the shoots and roots were then determined. After that, the contents of glycyrrhizic acid and flavonoids were determined by main roots and lateral roots separately. Chlorophyll fluorescence parameters were measured before plants were harvested.

### AMF Root Colonization

To determine the extent of AMF colonization, the roots of liquorice were cut into small blocks (about 1 cm^3^ in dimension), and stained with Trypan Blue following a modification of the procedure described by Phillips and Hayman (Koske and Gemma, 1989). AMF colonization in the root of liquorice was determined using the method described by Giovannetti and Mosse (1980).

### Chlorophyll Fluorescence Measurement

Chlorophyll fluorescence parameters of the two uppermost leaves of liquorice were measured at room temperature (25°C) using a dual-PAM-100 device (Heinz Walz, Effeltrich, Germany) following the protocols described by Ritchie and Bunthawin (2010). Prior to the measurement, the plants were kept in the dark for a minimum of 30 min, after which the minimal fluorescence in the dark-adapted state (F_0_) was recorded. A saturating pulse of irradiation (2 mmol m^-2^ s^-1^) for 3 s was then administered to measure the maximal fluorescence in the dark-adapted state (*F*_m_) (Gong et al., 2013). The leaves were then placed under actinic light (300 μmol m^-2^ s^-1^) to determine the maximal fluorescence (*F*_m_′), the minimal fluorescence in the light-adapted state (*F*_0_′) and the steady-state fluorescence (Fs). We calculated chlorophyll fluorescence parameters (*F*_v_/*F*_m_), Y(II), Y(NO), and Y(NPQ) following the description by Zai et al. (2012) and Tao (2014).

### Root System Measurement

The roots of cultivated liquorice were scanned with an Epson Expression/STD 4800 scanner (Seiko Epson Corporation, Nagano, Japan), and WinRHIZO image analysis software (Regent Instruments Inc., Quebec, QC, Canada) was used to derive the root length, surface area, volume, average diameter, the numbers of tips and forks, and the distribution of root lengths.

### Quantification of Bioactive Compounds in Root

Root materials were dried at 40°C for 2 days. An aliquot (0.1 g) of powdered root material (40 mesh) was extracted in 10 mL methanol/water (70:30) for 30 min in an ultrasonic bath at room temperature. The extract solution was cooled to room temperature and filtered through a 0.45 μm filter. A 10 μL aliquot of the filtrate was subjected to separation by high-performance liquid chromatography (HPLC) through a reverse phase C18 Symmetry^®^ column (4.0 mm × 250 mm, pore size of 3 μm; Waters Corp., Milford, MA, United States). The mobile phase comprised a gradient of deionized water: phosphoric acid (100: 0.020, *V/V*) and acetonitrile. The separation was operated in the gradient elution mode (Supplementary Table S1) at 25°C with a flow rate of 1.0 mL/min. Eluted compounds were detected spectrophotometrically at 276, 360, 248, and 370 nm using a 996 PDA photodiode array detector (Waters Corp., Milford, MA, United States). Glycyrrhizic acid, liquiritin, isoliquiritin, isoliquiritigenin, and licochalcone were purchased from China National Institutes for Food and Drug Control. Stock solutions were diluted with 70% aqueous methanol to appropriate concentrations for calibration purposes. (Zhang et al., 2013)

### Mineral Content Analysis of Soil and Plant Tissues

The dried plant tissue or soil samples (0.2 g) were digested in 10 mL mixture of perchloric acid (12.7 mol/L), sulfuric acid (18mol/L), and water (10:1:2) using the Mars 6 microwave reaction system (CEM Corporation, Matthews, NC, United States) until a clear liquid was obtained. The contents of total N, P, K in the samples were routinely analyzed, i.e., Kjeldahl method for total N, vanadium molybdate blue colorimetric method for total P, flame photometry for total K (Bao, 2000). The contents of Ca, Mg, Zn, Cu, Fe, and Mn were quantified by ICP-MS (Thermo Fisher Scientific Inc., Milford, MA, United States) (Gorecka et al., 2006).

Specifically, hydrolyzable N, available P, available K in soil was quantified using alkaline hydrolysis-diffusion method, ammonium acetate extraction-flame photometry, sodium bicarbonate extraction-vanadium molybdate blue colorimetric method, respectively. Available Ca, Mg were extracted with ammonium acetate; available Zn, Cu, Fe, and Mn were extracted with diethylenetriamine pentaacetic acid (Bao, 2000).

### Statistical Analysis

All the data were analyzed using the Statistical Package for Social Sciences (SPSS, version 18.0). Independent two-sample *t*-test was used to determine the statistical significance between the results of inoculated plants (GM) and non-inoculation control (CK; alpha = 0.05).

## Results

### Effect of *G. mosseae* Inoculation on Plant Growth

**FIGURE 2:**
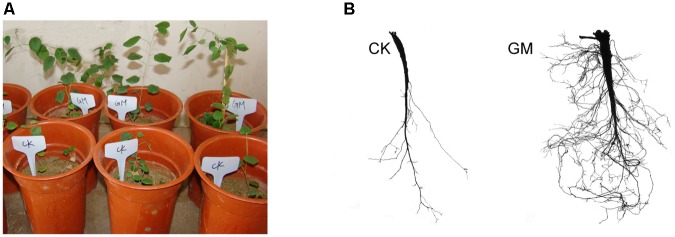
*Glomus mosseae* inoculated (GM) and non-inoculated liquorice plants (CK) in nutrients-deficient medium and their root system **(A)** plants **(B)** root system.

**FIGURE 3:**
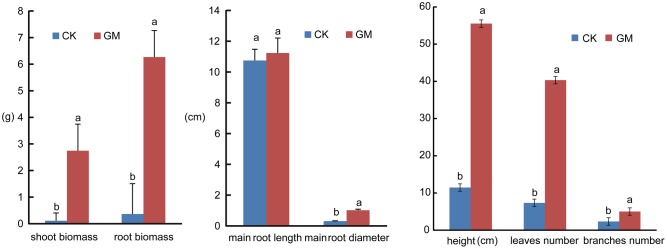
Shoot and root biomass accumulation, plant height, leaf number, branch number and root diameter of inoculated and non-inoculated liquorice plants. GM: plants inoculated with *G. mosseae*; CK: non-inoculated plants. Bars labeled with a different lowercase letter indicate a significantly different level of performance (*P* ≤ 0.05).

Among the liquorice plants treated with GM, 85% ± 3% were successfully colonized by *G. mosseae* as shown by the presence of hyphae, arbuscules, and vesicles (data not shown). No AMF fungal structures were observed in the roots of the plants with CK treatment. *G. mosseae* improve liquorice plant growth (**Figure 2**) and significantly increased the shoot biomass, root^∗^ biomass, plant height, leaf number, branch number, and root diameter by 25-, 17-, 5-, 6-, 2.5-, and 3-folds, respectively (**Figure 3**). The presence of AMF increased the projected area of the root system and the root surface area by 66.0 and 22.6%, respectively. The root volume and the overall length and the numbers of tips and forks were increased by 8.8-, 9.2-, 5.9-, and 26.8-folds, respectively (**Table 1**). The correlation coefficients of shoot biomass with the root system’s projected area, the roots’ surface area, mean diameter, volume, and the number of tips and forks were 0.76, 0.64, 0.78, 0.71, 0.64, and 0.76, respectively; the correlation coefficients of root biomass with these root parameters were 0.57, 0.57, 0.67, 0.75, 0.55, and 0.64, respectively.

**Table 1:** Effects of *G. mosseae* inoculation on liquorice root system.

Treatment	Projected area (cm^2^)	Surface area (cm^2^)	Average diameter (mm)	Volume (cm^3^)	Tips	Forks	Total length (cm)
CK	8.7 ± 0.6^b^	17.5 ± 1.4^b^	0.41 ± 0.04^a^	0.11 ± 0.02^b^	525 ± 201^b^	151 ± 13^b^	87 ± 8^b^
GM	14.4 ± 1.0^a^	21.5 ± 0.6^a^	0.40 ± 0.02^a^	1.08 ± 0.16^a^	3600 ± 627^a^	4189 ± 972^a^	886 ± 133^a^

*GM: plants inoculated with *G. mosseae*; CK: non-inoculated plants. Within each column, values associated with a different lowercase letter differ from one another significantly (*P* ≤ 0.05)*.

### Effects of *G. mosseae* on Chlorophyll Fluorescence

The presence of *G. mosseae* decreased the photosynthesis-related parameter Y(NPQ) by 44%, while increased *F*_v_/*F*_m_,^∗^ ETR, ^∗^and^∗^ *Y*(II) by 13.4, 34.7, and 31.3%, respectively (**Table 2**). There was no difference in *Y*(NO) between GM and CK treatment groups.

**Table 2:** Effects of *G. mosseae* Inoculation on chlorophyll fluorescence parameters of liquorice seedlings.

Treatment	*F*_v_/*F*_m_	*Y*(II)	*Y*(NO)	*Y*(NPQ)	ETR
CK	0.67 ± 0.02^b^	0.48 ± 0.03^b^	0.34 ± 0.02^a^	0.18 ± 0.03^a^	32.4 ± 1.9^b^
GM	0.76 ± 0.02^a^	0.63 ± 0.02^a^	0.32 ± 0.03^a^	0.10 ± 0.02^b^	44 ± 4^a^

*GM: plants inoculated with *G. mosseae*; CK: non-inoculated plants. Within each column, values associated with a different lowercase letter differ from one another significantly (*P* ≤ 0.05)*.

### Effect of *G. mosseae* Inoculation on Active Ingredients Contents in Roots

**FIGURE 4:**
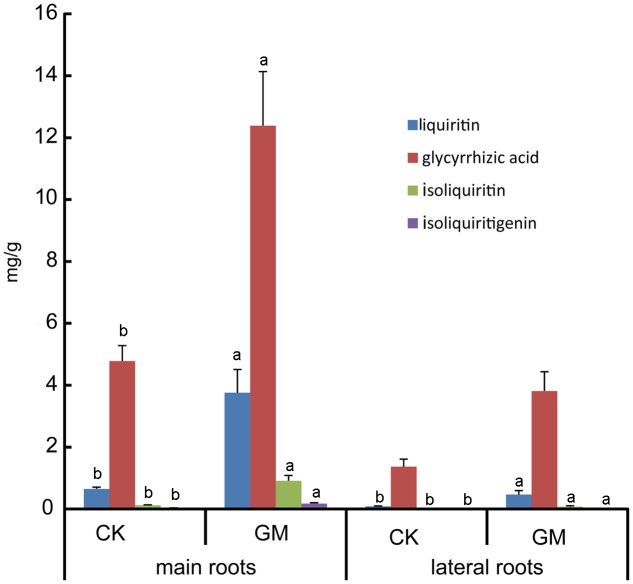
Bioactive constituents accumulation in inoculated and non-inoculated liquorice plants. GM: plants inoculated with *G. mosseae*; CK: non-inoculated plants. Bars labeled with a different lowercase letter indicate a significantly different level of performance (*P* ≤ 0.05).

Glycyrrhizic acid was the most prominent compound in liquorice root, whereas licochalcone was not detectable. *G. mosseae* inoculation increased the contents of glycyrrhizic acid, liquiritin, isoliquiritin, and isoliquiritigenin in the main root by 1.6-, 4.8-, 6.5-, and 4.4-folds, respectively, and increased glycyrrhizic acid and liquiritin contents in the lateral roots by 1.8- and 4.5-folds, respectively, as compared with those in CK-treated plants. Both isoliquiritin and isoliquiritigenin were detected in the lateral roots of GM-treated plants, but neither of these compounds was found in the lateral roots of CK-treated plants. The contents of glycyrrhizic acid, liquiritin, isoliquiritin, and isoliquiritigenin were all higher in the main root than in the lateral roots irrespective of the treatment of the plants (**Table 3** and **Figure 4**).

**Table 3:** Effects of *G. mosseae* inoculation on the contents of four bioactive constituents in the root system.

Part	Treatment	Glycyrrhizic acid (mg/g)	Liquiritin (mg/g)	Isoliquiritin (mg/g)	Isoliquiritigenin (mg/g)
Main root	CK	4.8 ± 0.4^b^	0.65 ± 0.06^b^	0.12 ± 0.02^b^	0.033 ± 0.004^b^
	GM	12.4 ± 1.8^a^	3.76 ± 0.08^a^	0.91 ± 0.17^a^	0.18 ± 0.03^a^
Lateral roots	CK	1.37 ± 0.25	0.085 ± 0.018^b^	0^b^	0^b^
	GM	3.8 ± 0.7	0.47 ± 0.13^a^	0.07 ± 0.03^a^	0.022 ± 0.004^a^

*GM: plants inoculated with *G. mosseae*; CK: non-inoculated plants. Within each column, values associated with a different lowercase letter differ from one another significantly (*P* ≤ 0.05)*.

### Effect of *G. mosseae* Inoculation on Plant and Soil Mineral Nutrient Status

In the plants with AMF treatment, the shoot and root P concentrations were raised by 62.5 and 138.9% and K concentration by 96.8 and 55.9%, respectively. The roots of GM-treated plants also showed a 36.7% increase in Ca content. The concentration of Mg in both the roots and shoots of the plant was reduced, while Zn, Mn, and Ca concentrations were lowered in the shoots. GM treatment induced no significant changes in root or shoot N and Cu contents or in root Zn and Mn contents of the plants, although their total accumulative quantities were higher in GM group (**Table 4**).

**Table 4:** Effects of *G. mosseae* inoculation on mineral nutrients in liquorice shoot and root.

Organ	Treatment	Total N (g/kg)	Total P (g/kg)	Total K (g/kg)	Total Cu (mg/kg)	Total Zn (mg/kg)	Total Mn (mg/kg)	Total Ca (g/kg)	Total Mg (g/kg)
Shoot	CK	14.8 ± 2.2^a^	0.64 ± 0.11^b^	10.0 ± 0.9^b^	7.9 ± 1.5^a^	35 ± 4^a^	59 ± 11^a^	6.5 ± 0.6^a^	3.1 ± 0.7^a^
	GM	15.6 ± 2.7^a^	1.04 ± 0.07^a^	19.7 ± 0.7^a^	7.5 ± 1.6^a^	20.1 ± 2.1^b^	17 ± 4^b^	2.66 ± 0.08^b^	0.95 ± 0.13^b^
Root	CK	19.4 ± 1.4^a^	0.36 ± 0.01^b^	2.99 ± 0.20^b^	5.9 ± 0.5^a^	20 ± 4^a^	15.7 ± 2.9^a^	1.77 ± 0.20^b^	1.72 ± 0.17^a^
	GM	17.5 ± 0.9^a^	0.86 ± 0.13^a^	4.66 ± 0.26^a^	5.4 ± 0.4^a^	25.5 ± 1.6^a^	12 ± 4^a^	2.42 ± 0.19^a^	0.87 ± 0.08^b^

*GM: plants inoculated with *G. mosseae*; CK: non-inoculated plants. Within each column, values associated with a different lowercase letter differ from one another significantly (*P* ≤ 0.05)*.

Inoculation with *G. mosseae* decreased the overall level in the rhizosphere soil of P and K as well as the levels of available P and K, but increased the levels of available Mg, Cu, Zn and Mn. In the soil samples, the presence of AMF decreased the contents of the total and available P contents by 53.5 and 34.5% and K contents by 41.0 and 51.2%, respectively, and increased Mg, Cu, Zn, and Mn contents by 27.3, 25.0, 34.9, and 46.0%, respectively (**Table 5**).

**Table 5:** Effects of *G. mosseae* inoculation on mineral nutrients in soil.

Treatment	Organic matter (g/kg)	Total N (%)	Total P (%)	Total K (%)	Hydrolyzable N (mg/kg)	Available P (mg/kg)	Available K (mg/kg)	Available Ca (mg/kg)	Available Mg (mg/kg)	Available Cu (mg/kg)	Available Zn (mg/kg)	Available Mn (mg/kg)	Available Fe (mg/kg)
CK	4.1 ± 0.4^a^	0.025 ± 0.012^a^	0.042 ± 0.001^a^	1.72 ± 0.11^a^	23 ± 4^a^	6.31 ± 0.18^a^	88 ± 6^a^	3908 ± 89^a^	183 ± 9^b^	0.36 ± 0.04^b^	0.30 ± 0.04^b^	1.76 ± 0.11^b^	5.88 ± 0.16^a^
GM	4.0 ± 0.5^a^	0.016 ± 0.001^a^	0.027 ± 0.001^b^	1.22 ± 0.07^b^	17.8 ± 2.1^a^	4.69 ± 0.22^b^	58 ± 5^b^	4094 ± 46^a^	233 ± 4^a^	0.48 ± 0.04^a^	0.407 ± 0.021^a^	2.57 ± 0.24^a^	6.02 ± 0.11^a^

*GM: plants inoculated with *G. mosseae*; CK: non-inoculated plants. Within each column, values associated with a different lowercase letter differ from one another significantly (*P* ≤ 0.05)*.

## Discussion

It is well established that AMFs can act as symbionts to promote the growth and productivity of the host plant (Shahabivand et al., 2012; Colla et al., 2015; Palencia et al., 2015; Yang et al., 2015). In our experiment, we prepared a nutrient deficient medium for liquorice growth by mixing paddy soil with river sand and pumice. All nutrients in the medium were in severe deficiency (Tian and Gao, 2005; Wang et al., 2013). According to a previous research, for 1000 Kg biomass accumulation of liquorice whole plant, there’s a need of 20.9 Kg N, 11.5 Kg P_2_O_5_ and 7.2 Kg K_2_O; and the P absorption increases significantly at the later growth stage (Cheng et al., 2005). The medium we prepared couldn’t meet these needs. Thus, the growth of liquorice plants in the experiment was under an obvious nutrient stress, especially P.

The major benefit of AMF derives from their effect on the host root system (Gutjahr et al., 2009; Vos et al., 2013). Inoculated seedlings produce longer, larger roots, which are thus able to exploit a larger volume of soil (Gutjahr et al., 2009). Mycorrhizal alterations of the root system architecture were AMF species-dependent. In the case of peach (*Prunus persica*), inoculation with *G. mosseae* and *G. versiforme* markedly increased the root length, root projected area, root surface area and root volume (Wu et al., 2011). Our results showed that the provision of *G. mosseae* effectively enhanced the biomass accumulation of liquorice plants, and markedly improved the host’s root system in terms of the projected area, the roots’ surface area, mean diameter, length and volume, and the number of tips and forks, which was consistent with previous reports (Atkinson et al., 2003; Liu et al., 2007; Orfanoudakis et al., 2010; Orujeia et al., 2013). The root system^∗^ architecture is critical in determining the capacity of plants to efficiently explore the soil (de Dorlodot et al., 2007). Here we demonstrate a positive correlation between the extensiveness of the root system and the accumulation of biomass. The plants provided with AMF (GM treatment) grew more rapidly than CK-treated plants with a more extensive root system, which facilitates nutrient uptake from the soil (Koske and Gemma, 1989; Olesniewicz and Thomas, 1999).

The presence of AMF has been shown to enhance the availability of certain soil micronutrients (Subramanian et al., 2013). We found that inoculation of *G. mosseae* increased the contents of available Mg, Fe, Cu, and Zn in the soil to promote their uptake by the plant. Kaya et al. (2009) reported that mycorrhizae helped to acidify the rhizosphere and solubilize certain tightly bound residual forms of Zn to allow more efficient transport of such metal ions via the symbionts’ hyphae to the host root. Available P and K in the soil were depleted in the pots with *G. mosseae* inoculation, probably due to their markedly enhanced uptake by the host plant, a phenomenon observed by previous researchers as well (Liu et al., 2007; Orujeia et al., 2013). As a result of the higher root and shoot biomass induced by *G. mosseae* inoculation, the concentration of Mg in both the roots and shoots of the plant was reduced, and for the same reason, Zn, Mn, and Ca concentrations were lowered in the shoots.

Biomass accumulation is strongly related to photosynthetic activity of the plant, and chlorophyll fluorescence parameters have been established as accurate predictors of photosynthetic ability and energy conversion efficiency (Dumlao et al., 2012). Photosynthesis is commonly reduced by nutrient deficiency (Kirschbaum and Tompkins, 1990; Turnbull et al., 2007). AMF symbiosis can reverse the unfavorable growth status of host plants under nutrient stresses through improving photosynthesis. According to Abdel-Fattah et al. (2014) the presence of *Glomus* symbionts improves growth and nutrition of the soybean plant through increasing photosynthesis in leaves, particularly at low P in soil. *G. mosseae* inoculation appeared to protect the photosystem II in beach plum (*P. maritima*) individuals subjected to salinity stress, enhance the efficiency of primary light energy conversion, and improve the response of photosynthesis (Zai et al., 2012). In *Medicago truncatula*, the provision of AMF is associated with an increase in leaf surface area, thereby raising the plant’s photosynthetic capacity without increasing the photosynthetic activity per unit leaf area (Adolfsson et al., 2015). In this study, inoculation with *G. mosseae* suppressed the photosynthesis-related parameter Y(NPQ), but promoted *F*_v_/*F*_m_, ETR and Y(II). The plants grown in the absence of AMF showed an *F*_v_/*F*_m_ of only 0.70 (**Table 2**), which was lower than the normal level, suggesting that photosynthesis in these plants was not operating at its full capacity (Klamkowski et al., 2009). In the presence of *G. mosseae*, *F*_v_/*F*_m_ was raised by 13%, which resulted in an about 35% increase in ETR. As the energy captured by chlorophyll can either be assimilated or dissipated as heat or re-emitted light, we presume that inoculation with *G. mosseae* bolstered energy assimilation [as measured by the parameter Y(II)] while reducing energy dissipation [Y(NO) and Y(NPQ)].

In the plants with AMF treatment, the shoot and root P and K concentrations were significantly raised. K is the most abundant univalent cation in plant cells and plays a significant part in regulating stomatal function (MacRobbie, 1998); its deficiency reduces photosynthesis by decreasing stomatal conductance (Peaslee and Moss, 1968; Terry and Ulrich, 1973). Increased K uptake accompanied with *G. mosseae* inoculation provided sufficient K supply for normal stomatal function, and thus might enhance photosynthesis of liquorice plant. In a recent study on cotton, it was found that K application significantly promoted the net photosynthetic rate of unit chlorophyll and increased significantly the *F*_v_/*F*_m_, Φ_PSII_ and qP (Hu et al., 2016). The increased uptake of P might also contribute to the improvement of photosynthesis. Similar phenomenon had been observed by Warren that higher concentrations of P in the nutrient solution led to significantly faster maximum net photosynthetic rate in *Eucalyptus globulus* (Warren, 2011).

Inoculation with AMF has been demonstrated to affect the production of the secondary metabolites, predominantly phenolics and terpenes (Pedone-Bonfim et al., 2015). In this study, we found that the provision of *G. mosseae* enhanced the capacity of liquorice root to produce flavonoids (with an increase by 4.4- to 4.8-folds) but to a lesser extent to enhance triterpenoid saponin content (1.6-folds). Both isoliquiritin and isoliquiritigenin were detected in the lateral roots of inoculated plants but not in those of non-inoculated plants. The increased glycyrrhizic acid accumulation in the roots of licorice plantlets inoculated with mycorrhizal fungi was proposed to be mainly due to the induction of plant defense system (Orujeia et al., 2013). In many cases of fungi colonization, flavonoids are specifically induced by symbionts and pathogens, and respond to purified signaling molecules from these organisms. The flavonoid pathway to synthesize specifically certain products has been suggested as an avenue to improve root-rhizosphere interactions (Hassan and Mathesius, 2012). The release of flavonoids into the rhizosphere can help to protect the host against a number of pests and diseases (Ndakidemi and Dakora, 2003) and regulate root growth and functions (Buer et al., 2010).

Flavonoids exudation can also affect nutrient availability through soil chemical changes, such as N, P, Fe, Mn, Cu, etc. (Cesco et al., 2010). This may partly be the reason we found increased P, K in the plant and available Fe, Mn, Cu in the soil. In turn, the increased nutrient absorption seems to improve the flavonoids accumulation. For example, in American skullcap (*Scutellaria lateriflora*), the yield of scutellarein, baicalin, baicalein, and chrysin increased with addition of P, and a linear response to K fertilization was observed for scutellarein concentration (Shiwakoti et al., 2016). Similarly, K and P application significantly increased liquirtin and glycyrrhizic acid content in the root of liquorice cultivated for 2 or 3 years in the fields (Fu et al., 2013). The increase of bioactive compounds accumulation and the increase of P and K uptake in *G. mosseae* inoculated liquorice might be of mutual promotion.

The contents of four major flavonoid constituents investigated in the main root were all higher than those in the lateral roots (**Table 3**); it was consistent with the finding that the secondary metabolites in liquorice were not evenly distributed throughout the plant (Guo et al., 2014). The failure of isoliquiritin and isoliquiritigenin detection in lateral roots of non-inoculated liquorice may due to its poor growth, of which the lateral roots were thin and small. Isoliquiritin and isoliquiritigenin might not be synthesized by the roots in such a state, or the amount is too little to be detected.

## Conclusion

The inoculation of liquorice plants with *G. mosseae* is beneficial, particularly for plants in nutrient-deficient soils. *G. mosseae* inoculation can facilitate the absorption of nutrients by improving the structure of root system, so as to raise photosynthesis efficiency, promote the growth of plants and increase the bioactive components.

## Author Contributions

GY, PL, YS, and XZ carried out experiments; MC and HQ analyzed data; MC and ZC completed the manuscript. MC, ZC, and LH designed the experiments.

## Conflict of Interest Statement

The authors declare that the research was conducted in the absence of any commercial or financial relationships that could be construed as a potential conflict of interest.

## Footnotes

1 http://foc.eflora.cn/
