# Supplementary material for: Glomus mosseae Inoculation Improves the Root System Architecture, Photosynthetic Efficiency and Flavonoids Accumulation of Liquorice under Nutrient Stress
Source: Front Plant Sci. 2017 Jun 7;8:931. doi: 10.3389/fpls.2017.00931 (PMC5461296; doi:10.3389/fpls.2017.00931)
Supplement: Supplementary file 1 [file Table_1.DOCX]

**Table S1. HPLC Mobile Phase and** [**Gradient Elution**](http://dict.cnki.net/dict_result.aspx?searchword=梯度洗脱条件&tjType=sentence&style=&t=condition+of+gradient+elution)

| Time (min) | A (acetonitrile) | B (deionized water : phosphoric acid) |
| --- | --- | --- |
| 0.0 | 14% | 86% |
| 10.0 | 23% | 77% |
| 24.0 | 30% | 70% |
| 30.0 | 34% | 66% |
| 35.0 | 36% | 64% |
| 42.0 | 42% | 58% |
| 48.0 | 51% | 49% |
| 60.0 | 14% | 86% |
